# Genetic diversity of cucumber green mottle mosaic virus (CGMMV) infecting cucurbits

**DOI:** 10.1016/j.sjbs.2022.02.027

**Published:** 2022-02-19

**Authors:** Zohaib Asad, Muhammad Ashfaq, Naeem Iqbal, Fahed Parvaiz, Mirza Abid Mehmood, Akhtar Hameed, Amir Humayun Malik, Samah Bashir Kayani, Mohamed A. Al-Kahtani, Zubair Ahmad

**Affiliations:** a Department of Plant Pathology, PMAS- Arid Agriculture University Rawalpindi, Pakistan; b Plant Pathology, Institute of Plant Protection, MNS- University of Agriculture Multan, Pakistan; c Department of Biosciences, COMSATS University, Islamabad, Pakistan; d Former Biotechnology Specialist, Centre for Agriculture and Bioscience International (CABI), Park Road, Islamabad, Pakistan; e Department of Zoology, G. Associate College (W), Mumtazabad, Multan, Pakistan; f Biology Department, Faculty of Science, King Khalid University, P.O. Box 9004, Abha 61413, Saudi Arabia; g Research Center for Advanced Materials Science (RCAMS), King Khalid University, P.O. Box 9004, Abha 61413, Saudi Arabia; h Unit of Bee Research and Honey Production, Faculty of Science, King Khalid University, P.O. Box 9004, Abha 61413, Saudi Arabia; i Biology Department, College of Arts and Sciences, Zehran Junub, King Khalid University, P.O. Box 9004, Abha 61413, Saudi Arabia

**Keywords:** Coat protein, CGGMV, Selection pressure, Recombination

## Abstract

*Cucumber green mottle mosaic virus* (CGMMV), a well-known *Tobamovirus*, infects cucurbits across the globe. To determine its current status, molecular characterization, genetic recombination, gene flow and selection pressure, 10 districts from Punjab province of Pakistan were surveyed and a total of 2561 cucurbits samples were collected during 2019–2020. These samples were subjected to virus-specific double antibody sandwich-enzyme linked immunosorbent assay (DAS-ELISA) for the detection of CGMMV. The results revealed that viral disease was prevalent in all surveyed districts of Punjab with an overall 25.69% disease incidence. ELISA positive samples were further confirmed through RT-PCR and sequencing of coat protein (CP) cistron. Sequence analysis showed that the present studied CGMMV isolates have 96–99.5% nucleotide and 94.40–99.50% amino acid identities with those already available in GenBank. Phylogenetic analysis also revealed that understudied isolates were closely related with South Korean (AB369274) and Japanese (V01551) isolates and clustered in a separate clad. Sequence polymorphisms were observed in 663 bp of sequence within 31 CGMMV isolates covering complete CP gene. Total number of sites were 662, of which 610 and 52 sites were monomorphic and polymorphic (segregating), respectively. Of these polymorphic, 24 were singleton variable and 28 were parsimony informative. Overall nucleotide diversity (π) in all the understudied 31 isolates was 0.00010 while a total of 1 InDel event was observed and InDel Diversity (k) was 0.065. Haplotype diversity analysis revealed that there was a total 29 haplotypes with haplotype diversity (Hd) of 0.993458 in all the 31 isolates which provide evidence of less diversity among Pakistani isolates. The statistical analysis revealed the values 2.568, 5.31304 and 4.86698 of Tajima's D, Fu, & Li’s F* and D*, respectively, which witnessed the population of CGMMV was under balanced selection pressure.

## 1 Introduction

The family *Cucurcbitaceae* includes several edible and non-edible species that are being grown across the globe particularly in tropical regions warm temperature (Ali et al., 2014; Asad et al., 2022). Economically important species of the family *Cucurbitaceae* are cucumber (*Cucumis sativus*), bottle gourd (*Lagenaria siceraria*)*,* melon (*Cucumis melo L.*), sponge gourd (*Luffa acutangula*)*,* summer squash (*Cucurbita pepo*)*,* bitter gourd (*Momordica charantia*) and serpent gourd (*Cucumis melo* var. *flexuosus*)*,* (Bisognin, 2002; Dhillon et al., 2020). In Pakistan, cucurbits are grown throughout the country due to their nutritive as well as medicinal importance (Adams et al., 2011, Ali et al., 2014, Różyło et al., 2014 Al-Jahani and Cheikhousman, 2017; Ashfaq, et al., 2021a). The leading producers of cucurbits are Turkey, China, India, and the United States. In Pakistan, total area under cucurbits cultivation is 32,848 ha with an annual production of 357,064 tons (http://www.crs.agripunjab.gov.pk/). In Punjab province of Pakistan, total area under cucurbits cultivation is 20,433 ha and total annual production is 259,365 tons with an average of 12 tons/ ha (Anonymous, 2018–19). The average yield of cucurbit crops in Pakistan is comparatively low that is attributed to several biotic and abiotic factors (Asad et al., 2022). Among the biotic factors, plant viruses especially RNA viruses are major threat to vegetables production in general and cucurbits in particular (Kone et al., 2017; Ashfaq, et al., 2021a).

Traditional production technology adopted by the farmers, conducive environment as well as lack of awareness about virus disease epidemiology, poor management practices and use of uncertified seeds may result in higher incidence of viral diseases (Ali et al., 2004; Ahsan et al., 2020; Ashfaq et al., 2008; Ahsan et al., 2023, Ashfaq et al., 2021b). About 59 viruses from different taxonomic groups are known to infect cucurbits resulting in high yield losses with difficult to manage (Lecoq and Desbiez, 2012, Crespo et al., 2017 Ahmed et al., 2021). Among these viruses, *Cucumber green mottle mosaic virus* (CGMMV) is a most devastating virus that is known to cause clossal losses in cucurbits (Ali et al., 2004). CGMMV belongs to genus *Tobamovirus* and is a highly stable virus that can persist a long time even in the absence of any living host (Hull, 2013, Reingold et al., 2015) and more than four years in dry leaves (Mandal et al., 2008). Firstly, it was identified in cucumber from Great Britain by Ainsworth (1935) and subsequently reported from several Asian and European countries e.g., India (Capoor and Varma 1948), Netherlands (Van Koot and van Dorst, 1959), Japan (Komuro, 1971), Taiwan (Wang and Chen, 1985), Israel (Antignus et al., 1990), Korea (Lee et al., 1990), Saudi Arabia Al-Shahwan and Abdalla, 1992, Spain (Celix et al., 1996), Poland (Pospieszny et al., 1997), Greece (Varveri et al., 2002), Pakistan (Ali et al., 2004), China (Chen et al., 2006), Ukraine (Budzanivska et al., 2007) and Russia (Slavokhotova et al., 2007). Recent outbreak of CGMMV have also been reported in United States (Tian et al., 2014), Canada (Ling, Li, and Zhang, 2014), Spain (Crespo et al., 2017) and Australia (Tesoriero et al., 2015).

The *Tobamovirus* has positive-sense RNA genome and virions are helical (Lewandowski, 2000; Adams et al., 2012, Darzi et al., 2020). *Tobamovirus* RNA encodes coat protein (CP) of 17.5 kDa, replicase proteins of 130 and 180 kDa, and movement protein (MP) of 30 kDa. (Crespo et al., 2017). According to Ainsworth (1935) first strain of CGMMV was described as cucumber virus 3 (CV3) followed by cucumber virus 4 (CV4) in Japan, two cucumber strains (Inouye et al. 1967), watermelon strain (Komuro et al., 1968) and Yodo strain (Kitani et al., 1970). The characteristics symptoms produced by CGMMV in cucurbits are leaf mottling, mosaic, blistering, and stunted growth to severe distortion of fruit (Antignus et al. 2001). However, variation in symptoms produced by CGMMV in different cucurbits relies upon the infected plant species and strain involved in inducing infection (Mandal et al., 2008). Transmission of virus occurred mostly through infected seeds and prevail on seed surface as a surface contaminant (Lecoq and Desbiez, 2012). Irrigated water and farm equipment are also source of virus transmission (Li, Liu, and Gu, 2016). Mechanical transmission of virus through propagated material made this virus more catastrophic (Sui et al., 2019). Mechanical means of transmission include wounds created by farm equipment during handling of plant and contact of leaves (Li et al., 2016). To date, virus transmission is not observed by any insect vector however, in India only 10% virus transmission has been observed through cucumber leaf beetle, *Raphidopalpa faeveicollis* (Mandal et al., 2008) and pollinators viz. European honeybees (*Apis mellifera* L) (Darzi et al., 2018). Some weeds of families; *Apiaceae, Amaranthaceae, Lamiaceae, Boraginaceae, Solanaceae, Chenopodiaceae* and *Portulacaceae* also serve as virus reservoirs (Boubourakas et al., 2004, Shargil et al., 2017, Dombrovsky et al., 2017).

Highly stable genome and transmission through different ways makes this virus a threatening challenge across the world wherever cucurbits are being cultivated (Smith and Dombrovsky, 2019). Occurrence of the inoculum right from the seedling development results in a very high infection level and it is very challenging for plants to resist against infection in such conditions (Coutts and Jones, 2002). ELISA and Polymerase Chain Reaction (PCR) are most successful and versatile techniques for routine diagnosis of plant viruses (Boonham et al., 2014). The limited work regarding molecular characterization of CGMMV is only confined to Khyber Pakhtunkhwa (KPK) Province of Pakistan (Ali et al., 2014). The Punjab province is a main hub for production of agronomic and horticultural crops so the present study was planned to determine the incidence of CGMMV and its phylogenetic relationship as well as genetic diversity in Punjab, Pakistan.

## 2 Materials and methods

### 2.1 Field survey and sample collection

**Fig. 1:**
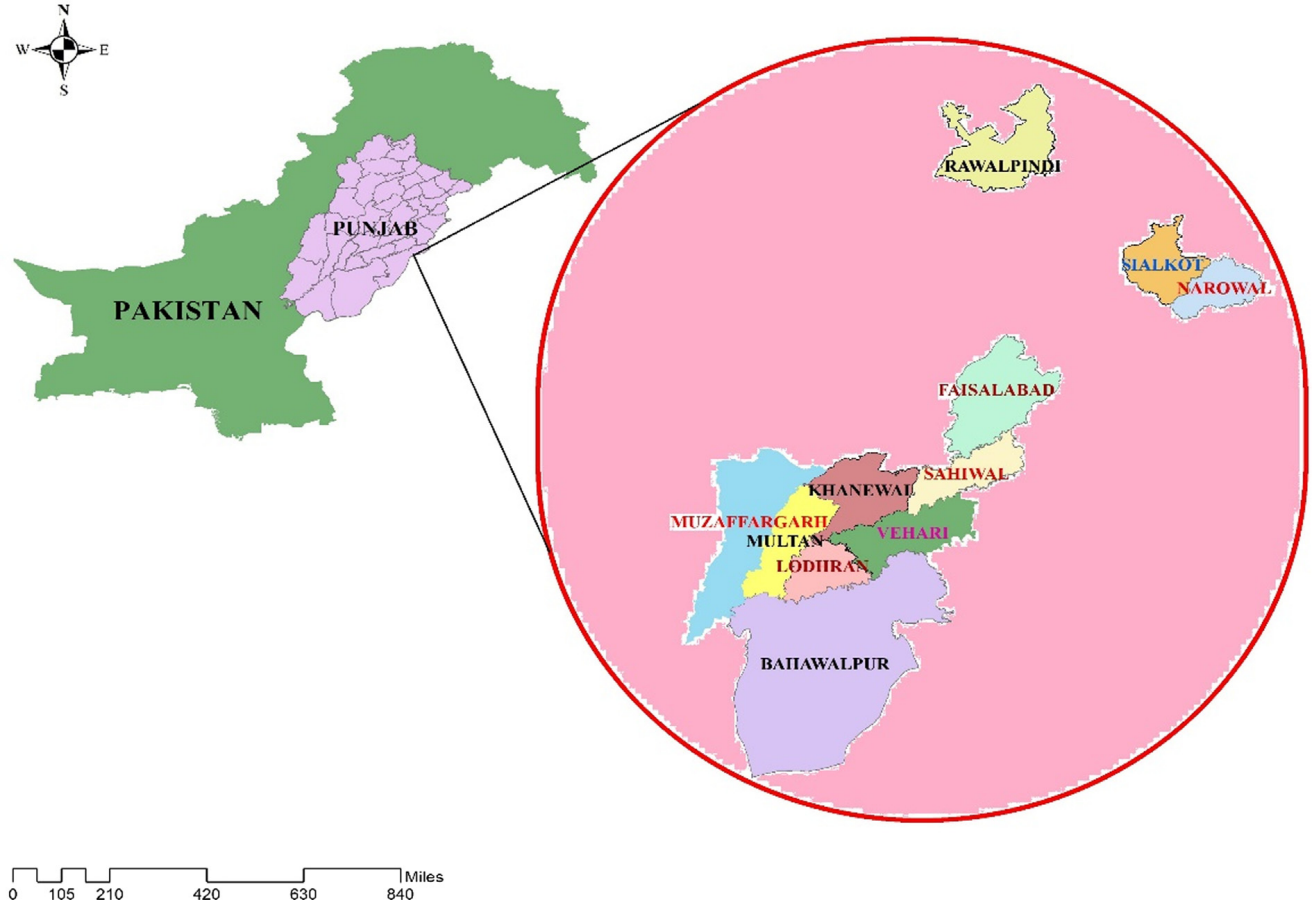
Map of under study districts of Punjab, Pakistan.

Diagnostic surveys were conducted in 2019 and 2020 in 10 districts of Punjab province, viz Rawalpindi, Sialkot, Faisalabad, Sahiwal, Khanewal, Vehari, Lodhran, Bahawalpur, Muzaffargarh and Multan (Fig. 1). A random stratified design (Khan et al., 2015) was adopted to check the incidence and distribution of CGMMV infecting cucurbits. Approximately 95–100 cucurbits fields were visited each year (total approximately 200 fields in two years) and 2561 samples from different cucurbit crops were collected in a diagonal survey of fields showing leaf mottling, mosaic, blistering, and stunted growth to severe distortion of fruit. To facilitate the revisit, all the locations were marked with Global Positioning System (GPS). All the collected samples were put into plastic zipper bags and kept on an ice-bucket and brought back to the Plant Virology Laboratory, MNS University of Agriculture, Multan. The samples were washed with tap water for removing superficial materials (dirt and other contamination) and divided into two slots. One slot was preserved and dried on silica gel for serological analysis and second was stored at -20 ℃ for further study.

### 2.2 Serodiagnosis of CGMMV

All samples were confirmed for the presence of CGMMV by using virus-specific DAS-ELISA as performed earlier (Clark and Adams, 1977). CGMMV-specific coating antibody (IgG) was diluted in coating buffer @ 1:200 and coating of ELISA plate wells was done using coating buffer at 200 μL/ well. ELISA plate was incubated overnight at 4^o^C in a moist chamber followed by three washing with TBS-Tween buffer at an interval of 5 min. CGMMV symptomatic leaf samples were homogenized (1:20) in an extraction buffer and sample sap was added @ 200 μL/ well. The plate was incubated overnight at 4^o^C followed by 3-4 washings at 5 min intervals and blot-dried on a paper towel. Then conjugated antibody (1:200) were added @ 200 μL/ well and incubated overnight at 4^o^C. Substrate buffer containing p-nitrophenyl phosphate (75 μg mL^-1^) was freshly prepared and added at 200 μL/ well followed by incubation at room temperature for 30 min. The absorbance values i.e., 405 nm were measured with an Automatic ELISA Reader (HER-480 HT Company (Ilford) Ltd, UK). Samples were considered positive for CGMMV infection when the ELISA absorbance value was equal to two times or higher than the average of the absorbance value of the healthy sample as well as the negative control (Ashfaq et al., 2014). Commercial positive and negative controls were used as a reference provided by Bioreba. The reaction was stopped by adding 3 M NaOH at 50 μL/ well. Relative disease incidence was calculated by using the following formula (Asad et al., 2022).

$$\mathrm{Disease}\;\mathrm{incidence}=\frac{\mathrm{Infected}\;\mathrm{samples}}{\mathrm{Total}\;\mathrm{samples}}\times 100$$

### 2.3 RT-PCR amplification and sequence analysis

Extraction of total RNA from the ELISA positive along with some healthy samples of cucurbits were performed using the TRIzol® Reagent (Life Technologies, Carlsbad, USA). Quantification of RNA was performed using Nanodrop (Thermo Scientific Co. USA) according to standard guideline of manufacturer. Working dilution of RNA at 500 ng/μL was used for synthesis of first strand complementary DNA (cDNA) using the Revert Aid, first strand cDNA synthesis Kit and CGMMVR-53 5′-TTG CAT GCT GGG CCC CTA CCC GGG GAA AG-3′ (Hongyun et al., 2008) as a virus-specific downstream reverse primer. The resultant cDNA (2 µL) was used for PCR amplification along with following PCR ingredients; DreamTaq Green PCR master mix (2X) (12.5 µL) (Thermo Fisher Scientific, USA Cat. No. K1081), virus-specific upstream primer (CGMMVF-52 5′-CCG AAT TCA TGG CTT ACA ATC CGA TCA C-3′ as well as CGMMVR-53 as a downstream primer and nuclease free water up to final reaction volume of 25 µL (Hongyun et al., 2008).

The amplification scheme was set as: initial denaturation for 3 min at 94℃ followed by 35 cycles: 30 s at 94℃, 53℃ for 45 s and 72℃ for 60 s, and final extension was done at 72℃ for 5-min. PCR products were analysed by electrophoresis with 1.0% (w/v) pre-stained agarose gel and visualized under Omega Fluor™ Plus Gel Documentation System (1149C42). The desired amplicons with 700 bp were purified using GeneJET PCR Purification Kit and cloned into pJET1.2/blunt cloning vector with chemically competent cells of *E. coli* strain XL1-Blue. Recombinant plasmid DNA was purified using the GeneJET Plasmid Miniprep Kit as per manufacturer's instructions. Digestion with restriction enzymes (*BglII*) confirmed the presence of an insert in transformants and positive clones were sequenced at Macrogen Inc. (North Korea) in both orientations. The obtained sequences were analysed using the National Center for Biotechnology Information; Basic Local Alignment Search Tool (NCBI BLAST) application and compared with different isolates of CGMMV reported from elsewhere in the world. The CGMMV nucleotide sequences identified in the present study were submitted to the database.

### 2.4 Phylogenetic study of complete CGMMV CP gene

CGMMV sequences identified in the present study were aligned with related CGMMV sequences retrieved from GenBank by using Clustal W embedded in MEGA7 software (Tamura et al., 2013). After aligning, their phylogenetic relationship and ancestral lineage was deduced using the Neighbor-Joining method with 1000 bootstrap replicates (to epitomize the taxa’s evolutionary history) in MEGA 7 software (Tamura et al., 2013). The nucleotide and amino acid sequence identity were calculated by Sequence Identity Matrix option in BioEdit v7.2.6.1 (Hall, 1999).

### 2.5 Analysis of recombination event and selection pressure

Aligned sequences consists of 13 Pakistani CGMMV and 18 other isolates reported elsewhere in the world were analysed using RDP4 to detect apparent recombinant events in the identified sequences of CGMMV isolates in Pakistan (Table 2) by applying the general tab with the default settings which implement all the available methods viz. RDP, GENECONV, BootScan, MaxChi, SiScan, Chimaera and 3SEQ for this purpose. The Nucleotide diversity (π), number of polymorphic (segregation site, S), insertion and deletions (InDel), haplotype diversity (Hd) and synonymous (Ka) and non-synonymous (Ks) rate of mutations, gene flow, genetic differentiation and neutrality within each group and defined region, statistical tests like F*st,* Z, Ks*, Snn, Tajima’s D, Fu, Li’s D* and F* were calculated using DnaSP v 6.12.03 (Fu and Li, 1993, Tajima, 1989, Rozas et al., 2017).

## 3 Results

### 3.1 Detection of virus through serological assay

Samples with mosaic, mottling and blistering type symptoms were confirmed positive for the infection of CGMMV using DAS-ELISA. The present study revealed that the CGMMV was prevalent in all cucurbits including watermelon, ridge gourd, cucumber, sponge gourd, pumpkin, bitter gourd, squash and melon in Punjab, Pakistan. Overall disease incidence was slightly higher i.e., 27.96%, in 2019 compared to 24.74% in 2020 (Table 1A, Table 1B). During 2019, the highest disease incidence of CGMMV was recorded in district Rawalpindi (39.39%) followed by district Khanewal (32.72%), Vehari (30.93%), Faisalabad (30.82%), Multan (29.86%), Sahiwal (24.26%), Bahawalpur (22.62%), Lodhran (22.41%) while least incidence was observed in district Muzaffargarh (21.56%, Table 1A). In 2020, relatively low viral incidence was recorded compared to 2019 with the highest mean virus incidence i.e., 32.84% was observed in Faisalabad while lowest incidence was recorded in district Lodhran (18.18%). Incidence of virus infection in other eight districts of Punjab was recorded as 30.82% in Rawalpindi, 25.73% in Sahiwal, 25.22% in Khanewal, 23.07% in Vehari, 22.48% in Multan, 21.80% in Bahawalpur, and 21.58% in Sialkot. Furthermore, the highest disease incidence (37.15%) was recorded on cucumber crop in 2019 while lowest disease incidence (21%) was observed on bitter gourd crop. In 2020, cucumber crop was observed as vulnerable to CGMMV with 32% disease incidence while bitter gourd replaced ridge gourd for lowest infection (19.17%) as shown in Table 1B.

**Table 1A:** Percentage disease incidence of CGMMV infecting cucurbits in Punjab, Pakistan during 2019.

Host	2019											
	Rawalpindi	Sialkot	Faisalabad	Sahiwal	Khanewal	Vehari	Lodhran	Bahawalpur	Muzaffargarh	Multan	T.I/T.C	% D.I
Water melon	6(15)	3(10)	8 (24)	5(13)	10(20)	8(18)	7(25)	5(20)	9(23)	5 (12)	66(1 8 0)	36.66%
Cucumber	9(19)	7(21)	6(16)	5(17)	7(20)	10(23)	6(16)	8(20)	4(12)	6(19)	68(1 8 3)	37.15%
Round gourd	6(20)	5(18)	4(12)	6(21)	5(11)	4(10)	2(8)	0(11)	2(8)	5(13)	39(1 3 2)	29.54%
Ridge gourd	8(20)	0(12)	3(10)	5(16)	1(10)	4(13)	3(12)	6(17)	3(13)	5(20)	38(1 4 3)	26.57%
Smooth gourd	6(13)	3(11)	8(21)	3(15)	4(11)	5(23)	0(15)	1(10)	1(5)	5(15)	36(1 3 9)	25.89%
Melon	5(10)	0(6)	2(6)	0(4)	2(5)	2(7)	2(8)	2(10)	0(8)	2(15)	17(79)	21.51%
Bitter gourd	5(12)	7(18)	6(25)	6(23)	3(13)	0(10)	0(12)	2(15)	0(11)	4(18)	33(1 5 7)	21.00%
Pumpkin	3(8)	0(7)	5(14)	3(12)	0(10)	4(15)	2(10)	4(19)	3(15)	7(22)	31(1 3 2)	23.48%
Squash	4(15)	2(15)	3(18)	0(15)	4(10)	6(20)	4(10)	3(15)	0(7)	4(10)	30(1 3 5)	22.22%
T.I (T.C)	52(1 3 2)	27(1 1 8)	45(1 4 6)	33(1 3 6)	36(1 1 0)	43(1 3 9)	26(1 1 6)	31(1 3 7)	22(1 0 2)	43(1 4 4)	358(1280)	
% D.I	39.39%	22.88%	30.82%	24.26%	32.72%	30.93%	22.41%	22.62%	21.56%	29.86%

**Table 1B:** Percentage disease incidence of CGMMV infecting cucurbits in Punjab, Pakistan during 2020.

Host	2020											
	Rawalpindi	Sialkot	Faisalabad	Sahiwal	Khanewal	Vehari	Lodhran	Bahawalpur	Muzaffargarh	Multan	T.I/T.C	%D.I
Water melon	4(18)	5(17)	7(15)	3(12)	8(19)	4(18)	4(20)	7(20)	6(19)	3(14)	51(1 7 2)	29.65%
Cucumber	10(20)	8(22)	5(18)	3(15)	4(18)	8(25)	3(12)	7(18)	5(15)	4(15)	57(1 7 8)	32%
Round gourd	5(22)	4(20)	5(15)	5(18)	3(12)	2(10)	3(7)	1(10)	4(10)	2(15)	34(1 3 9)	24.46%
Ridge gourd	5(22)	0(10)	4(12)	3(17)	2(12)	3(15)	2(12)	4(15)	2(15)	3(16)	28(1 4 6)	19.17%
Smooth gourd	5(10)	2(12)	10(20)	4(18)	3(12)	6(25)	2(15)	0(10)	2(8)	3(12)	37(1 4 2)	26.05%
Melon	3(8)	2(10)	2(7)	3(8)	1(7)	1(10)	0(10)	2(12)	2(10)	3(13)	19(95)	20%
Bitter gourd	4(10)	5(20)	5(23)	4(20)	0(7)	1(10)	1(10)	3(18)	2(10)	3(15)	28(1 4 3)	19.80%
Pumpkin	2(8)	1(10)	4(12)	6(15)	4(12)	3(12)	2(12)	2(17)	3(18)	5(19)	32(1 3 5)	23.70%
Squash	3(15)	3(18)	3(15)	4(13)	3(12)	5(18)	3(12)	2(13)	2(5)	3(10)	31(1 3 1)	23.66%
T.I (T.C)	41(1 3 3)	30(1 3 9)	45(1 3 7)	35(1 3 6)	28(1 1 1)	33(1 4 3)	20(1 1 0)	29(1 3 3)	28(1 1 0)	29(1 2 9)	317(1281)	
% D.I	30.82%	21.58%	32.84%	25.73%	25.22%	23.07%	18.18%	21.80%	25.45%	22.48%		

### 3.2 Molecular characterization and phylogenetic analysis

PCR results showed that CGMMV gene specific primers CGMMVF-52 and CGMMVR-53 amplified a specific fragment of 700 bp in all the selected ELISA positive samples representing each surveyed district and crop while no amplification was observed in case of negative control. A total of 13 CGMMV isolates were obtained and sequenced at Macrogen Korea. MegaBLAST analysis confirmed that amplified products were coat protein gene of CGMMV along with some portion of 3' UTRs. After sequence analysis, 13 isolates having accession numbers MW732114-MW732126 identified from cucumber, watermelon, melon, ridge gourd, sponge gourd, pumpkin, bitter gourd and squash were submitted to Genbank along with other previously reported isolates (Table 2).

**Fig. 2:**
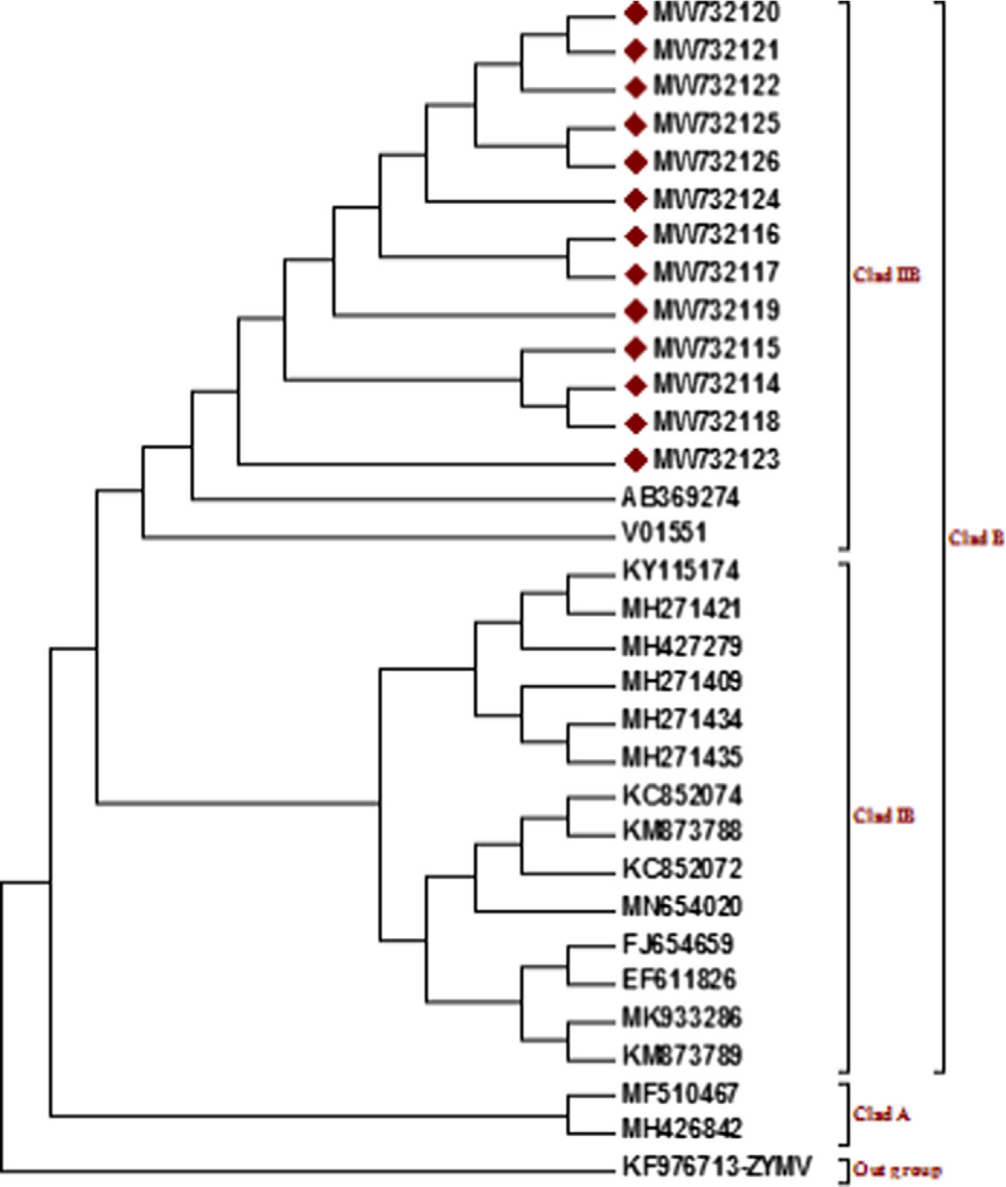
Evolutionary relationships of Pakistani CGMMV nucleotide sequences of CP gene with previously reported Genbank sequences by using Neighbor joining method.

Sequence identity matrix based on nucleotides and amino acids showed that all 13 isolates reported in current study shared 96.20%-99.5% similarity with isolates reported from other parts of the world and shared 98% to 99.5% similarity with each other (Table 3). Coat protein sequence based phylogenetic analysis of CGMMV isolates with the other reported sequences from China, Australia, USA, Greece, Canada, Netherland and Taiwan resulted in two main clads (A and B). Clad B is divided into two sub clads IB and IIB. In clad A, two Canadian isolates (MF510467 and MH426842) were present. In clad IB, isolates from China, Australia, Greece, Taiwan, Netherland and USA were present. In clad IIB, two isolates, one from South Korea (AB369274) other from Japan (V01551) and 13 isolates reported in this study were present while ZYMV used as an outgroup (Fig. 2).

**Table 2:** List of sequences used in identity matrix calculation and phylogenetic analysis.

Virus strains/ Isolate	Geographical region	Host	Accession number
WM strain	South Korea	*Nicotiana benthamiana*	AB369274
ON5	Canada	*Cucumis sativus*	MF510467
CGMMV-Ca	Canada	*Cucumis sativus*	MH426842
JSDT12	China	*Nicotiana benthamiana*	KC852072
CGMMV-NT	Australia	*Apis mellifera*	MH427279
HaiN12	China	*Nicotiana benthamiana*	KC852074
JSGY	China	*Lagenaria siceraria*	MN654020
CG003	USA	*Nicotiana benthamiana*	MH271409
BG	Taiwan	*Lagenaria siceraria*	FJ654659
Liaoning	China	Citrullus lanatus	EF611826
CG029	Greece	*Nicotiana benthamiana*	MH271434
WA-1	Australia	*Cucumis sativus*	KY115174
CG030	Greece	*Nicotiana benthamiana*	MH271435
TZ4	China	*Cucumis melo*	KM873788
GDLZ	China	*Lagenaria siceraria*	MK933286
DY13	China	*Cucumis melo*	KM873789
WM strain	Japan	*Cucumis sativus*	V01551
CG015	Netherland	*Nicotiana benthamiana*	MH271421
ZARWm	Pakistan	*Citrullus lanatus*	MW732114
ZAFWm	Pakistan	*Citrullus lanatus*	MW732115
ZAMRg	Pakistan	*Luffa acutangula*	MW732116
ZALPk	Pakistan	*Cucurbita pepo*	MW732117
ZASCu	Pakistan	*Cucumis sativus*	MW732118
ZANCu	Pakistan	*Cucumis sativus*	MW732119
ZABSg	Pakistan	*Benincasa hispida*	MW732120
ZAGRg	Pakistan	*Luffa aegyptiaca / Luffa cylindrica*	MW732121
ZAPMl	Pakistan	*Cucumis melo*	MW732122
ZAGSq	Pakistan	*Cucurbita maxima*	MW732123
ZACBg	Pakistan	*Lagenaria siceraria*	MW732124
ZACSq	Pakistan	*Cucurbita maxima*	MW732125
ZAVRg	Pakistan	*Luffa acutangula*	MW732126

**Table 3:** Comparison of Pakistani CGMMV CP gene nucleotides sequences to each other and retrieved Genbank sequences.

### 3.3 Selection pressure and recombination analysis

Sequence polymorphisms were observed in 663 bp of sequence within 31 CGMMV isolates covering complete CP gene. Total number of sites (excluding sites with missing data / gaps) were 662, of which monomorphic sites were 610 and 52 polymorphic (segregating) sites (S). Of these, 24 were singleton variable and 28 were parsimony informative. Overall nucleotide diversity (π) in all the studied 31 isolates was 0.00010 while a total of 1 InDel event was observed and InDel Diversity (k) was 0.065. Haplotype diversity analysis revealed that there are a total 29 haplotypes with haplotype diversity (Hd) of 0.993458 in all 31 isolates. This result provided evidence that Pakistani isolates have less diversity compared to the other reported isolates. Less π value also confirmed the phylogeny results as all 13 Pakistani isolates were present in the same clad. The Ks values range was 0.0030-0.9058 while the range of Ka was 0.00–0.8245 and total number of 358 mutations were observed. No Recombination event was detected in Pakistani CGMMV isolates. Moreover, all the positive values 2.568, 5.31304 and 4.86698 of Tajima's D, Fu, & Li’s F* and D*, respectively, (commonly used tests to recognize sequences that do not suit the neutral model in genetic drift and mutation equilibrium), weren’t statistically significant, respectively (Ramírez-soriano et al., 2008, Tajima, 1989) demonstrating that CGMMV population was under balanced selection pressure.

## 4 Discussion

Viruses infecting vegetables have always been a tremendous threat to sustainable vegetable production across the world (Amari et al., 2017, Moriones et al., 2017, Asad et al., 2022). In Pakistan, high incidence of vegetable viral diseases has already been reported by number of scientists (Ashfaq et al., 2015; Ashfaq and Ahsan, 2017; Hussain and Atiq, 2017, Tahir et al., 2017). Cucurbitaceae is the largest family among vegetables which includes cucumber, watermelon, ridge gourd, sponge gourd, melon, bitter gourd and these are vulnerable to a number of plant viruses (Rao et al., 2017). This study demonstrated the ubiquitous occurrence of CGMMV infecting cucurbit crops in the Punjab, Pakistan. According to our knowledge it is the first ever study regarding the occurrence, molecular characterization, genetic recombination and selection pressure of CGMMV in Punjab Pakistan.

CGMMV significantly affected the cucurbit crop yield and resulted in a variable symptoms depending upon the genotype/ variety (Mandal et al., 2008). Green mottling symptoms generally appeared on fruits and young leaves of cucumbers and consequently resulted in death of the plants. In young watermelon plants, mosaic and mottling type symptoms appeared and brown necrotic lesions developed on peduncles and stems. The foliage became wilted and bleached resulting into the premature death of runners or even whole plants. However, in mature plants, particularly in open-field conditions, foliage symptoms were fade. Malformation and internal flesh symptoms of sponginess and yellowing or dirty red discoloration were common in their fruits rendering them unmarketable. Initially mottling and mosaic type symptoms appeared on young melon leaves, later on faded away as the foliage matures. Fruits showed varying degree of mottling, malformation, and netting on their surface. Infected foliage in zucchini, squash, and pumpkin were asymptomatic or had leaf mottling and mosaic. The symptoms caused by CGMMV on cucurbits were similar as observed in previous studies (Varveri et al., 2002; Shim et al., 2006; Wu et al., 2011; Reingold et al., 2013; Ali et al., 2014; AUSVEG, 2016).

It was observed that the growers purchased seeds from the open market or used their domestic seeds which served as a primary source of viruses in general and tobamoviruses in particular. Being a *Tobamovirus*, CGMMV is usually transmitted via contact, seeds, field implements as well as poor phytosanitary measures that intensify the prevalence and incidence under *in vivo* conditions. Besides this, the population of different weeds in the field and adjacent areas also played a significant role in the higher incidence of viruses. The incidence results are in agreement with Ali et al. (2014). While the contrary results were also reported by other scientists Ellouze et al., 2020, Yoon et al., 2008.

Disease incidence varied from species to species and efficiency of vectors (Mandal et al., 2008, Li et al., 2016). Virus characterization, their genetic composition, recombination and mechanism of variation helped in the develpoment of transgenic plants resistant against viral diseases. It also aid in studying the CGMMV Pakistani isolates relatedness to CGMMV isolates reported from other parts of the world and from same geographical region. It also predict about new strain evolution and its adaptation to new hosts and geographical conditions. The nucleotide (nt) and amino acid (aa) sequences for the CP gene of the 13 CGMMV isolates showed low diversity when compared with other isolates reported from other parts of the world. All the 13 isolates shared 97.20–99.50% nt and 94–99.50% aa sequence similarity with each other. The nt and aa identities of CGMMV Pakistani isolates with other isolates reported from China, Australia, USA, Greece, Canada, Netherland, Japan and Taiwan were recorded as 96–99.5% and 94.40–99.50%, respectively. The present study nucleotide sequence analysis showed less conservative trend as compared to Yoon et al., 2008, Ali et al., 2014 where it was reported to have 98–99% similarity among CGMMV isolates.

Coat protein sequence based phylogenetic analysis of present study CGMMV isolates with isolates reported from China, Australia, USA, Greece, Canada, Netherland and Taiwan resulted in two main clads (A & B). Clad B is further divided into two sub clads IB and IIB. In clad A, two Canadian isolates were present. Isolates from China, Australia, Taiwan, Greece, Netherland and USA were present in clad IB while the present study 13 isolates along with one isolate each from South Korea and Japan were present in clad IIB. This study provided evidence that CGMMV Pak isolates that infect cucurbits most probably originate from South Asia. As an evolutionary process, variations occurs in the genetic makeup of organisms by addition of new alleles through gene flow or mutations (Zu et al., 2019). Positive values of Neutrality tests i.e. Tajima's D test, Fu, & Li’s F*, Fu, & Li’s D* revealed that CGMMV population is under balanced selection pressure and low frequency of variation was observed (Tajima, 1989, Fu and Li, 1993). Mutation, re-assortment and recombination are the main causes of genetic diversity in RNA viruses (Holmes, 2006, Akinyemi et al., 2016) which may result in the enclosure of discrete sequence components along with interchange, repetition or obliteration of existing viral elements. No genetic recombination event was observed among the CGMMV isolates identified in current study. Our finding deviates from the findings of Rao et al. (2017) who reported very little recombination event but recombination score was below 60% which reflected that isolate is not recombinant.

## 5 Conclusion

CGMMV is a notorious and devastating pathogen responsible for huge losses in cucurbit crops all over the world. In the present study, CGMMV was detected in almost all cucurbit crops grown in Punjab, Pakistan with an overall disease incidence of 26.35% during 2019–2020. Evolutionary distance and phylogenetic analysis of 13 CGMMV isolates revealed that all of these isolates have a close relationship with Japanese and South Korean isolates. High frequency of gene flow with lower nucleotide diversity was detected in CGMMV population. Positive values of statistical tests showed balanced selection pressure in understudied CGMMV population. The presence of this potentially destructive virus in Punjab represents an alarming situation for successful production of cucurbit crops. Based on the findings of present study, necessary strategies including resistant genotypes and integrated management approaches are recommended to prevent the widespread occurrence of this virus.

## Declaration of Competing Interest

The authors declare that they have no known competing financial interests or personal relationships that could have appeared to influence the work reported in this paper.
